# Judiciary in Democracy: Alignment and Disconnect

**DOI:** 10.1007/978-3-030-63143-7_7

**Published:** 2020-12-15

**Authors:** Frans van Dijk

**Affiliations:** Faculty of Law, Economics and Organization, Utrecht University, Utrecht, The Netherlands

**Keywords:** Alignment, Disconnect, Judiciary, Democracy, Trias politica

## Abstract

In this last Chapter, the consequences of differences in perceptions are examined. Two concepts are used: the lack of alignment and—more extreme—the disconnect between judiciary and society. Ranking countries by trust in the judiciary, in the lowest 20% there is a disconnect of judiciary and society, in the 20% around the median and in the highest 20% there is lack of alignment. Disconnect and lack of alignment seem to be self-perpetuating, as judges do not perceive the state of independence as problematic. Indications are that even a disconnect does not reduce the use of the civil courts, but that it leads citizens to avoid administrative law procedures. A disconnect weakens the position of the judiciary within the trias politica. This reinforces the complicated relationship between the judiciary and the other state powers. Where the other state powers see an increasing influence of the judiciary, the judiciary sees its own independence endangered. These perspectives clash. For the judiciary the way out is to focus on access to justice as an alternative perspective. By addressing the urgent legal needs of citizens, the judiciary has the potential to improve its alignment with society and its position within the trias politica.

## Definition of Lack of Alignment and Disconnect

In Chapter 10.1007/978-3-030-63143-7_3 it is shown that, while the perceptions of the independence of judiciary by relevant groups are correlated across countries, their perceptions differ in level. Who rates the judicial system on this crucial dimension, makes a huge difference. The perceptions by judges of their own independence are more positive than their perceptions of the independence of the judges in general in their country, in turn these perceptions are more positive than the perceptions of lawyers about judicial independence and these again are (much) more positive than the perceptions of the general public and that of companies. Also, level of education has an impact on perceptions with citizens with low education being less positive about judicial independence for medium and high levels of independence. While the data about court users is scarce and contradictory, it seems that experience lowers the perception of independence further. This is consistent with research for the US that suggests that for most people going to court is losing control over their situation, and that this loss of control as such leads to a negative experience (Benesh 2006). In addition it was shown in Chapter 10.1007/978-3-030-63143-7_3 that winning or losing plays a role, where losing is likely to get greater weight in the minds of people than winning, resulting on balance in a negative effect on perceived independence.

In Chapter 10.1007/978-3-030-63143-7_4 it is shown for a smaller set of countries that citizens who act as lay judges have perceptions about the independence of lay judges and of professional judges that are at the same level as the perceptions of professional judges. Their perceptions are much more positive than those of the highly educated general public. In the comparison of citizens as lay judges and as parties, judging versus being judged is likely to play an important role, and in the comparison of lay judges and the general public direct experience versus indirect information. Large differences in the perceptions of independence by judges including lay judges and the perceptions of the public in general and the public as parties reflect a lack of alignment between judiciary and society. A lack of alignment may turn into a disconnect when judges are (highly) positive about their independence and the public (highly) negative. In that case judges and those (potentially) being judged differ fundamentally on the actual realization of the core value of the judiciary. For citizens this is not (only) an abstract matter about the division of powers within the state, but about getting a fair trial. As discussed in Chapter 10.1007/978-3-030-63143-7_2, independence is a key aspect of procedural justice. Perceptions of independence, however, do not constitute the full picture.

Low levels of perceived judicial independence by the public may go together and may be exacerbated by lack of respect for independence by court users (parties, lawyers, prosecutors), the political system ( parliament, government and media) and/or leaders of the judiciary (highest courts, governance bodies). Chapter 10.1007/978-3-030-63143-7_5 examines the perceptions of judges about the respect their independence receives from these groups. It is found that, according to the respondents, internal leaders respect the independence of the judges most, court users less so but still a lot, and the political system least. It is also shown that according to the judges respect for independence of the respective groups is based on different aspects of independence, related to the influence sphere of the respective actors. For instance, as to the parties, their respect is connected to corruption and inappropriate pressure, while for government and parliament the connection is with case load ( budget) and the implementation of judgments that go against political interests. Combining perceptions about independence and respect for independence, a disconnect between judiciary and society is defined here as the combination of (1) positive perceptions of judicial independence by judges and negative perceptions by the general public, and (2) lack of respect for independence by the court users and the political system.

Chapter 10.1007/978-3-030-63143-7_6 incorporates trust in the framework. As discussed in Chapter 10.1007/978-3-030-63143-7_6, trust is a broad concept that can be applied to any function or organization. It is much less specific than independence, and precisely for that reason allows comparisons across institutions. For the judiciary it was shown that trust and independence are highly correlated to such an extent that trust in the judiciary equals trust in the independence of the judiciary. It was argued that independence or—more precisely— autonomy plays a role in government in general. Government consists of highly political policy making and more neutral policy implementation which is often executed by seperate organizations such as more or less autonomous agencies (see Chapter 10.1007/978-3-030-63143-7_2). For these latter organizations, impartiality and independence are important values, and they may even end up in court if they do not abide by these values. Trust in this public administration is likely to have a similar content as trust in the judiciary. For the political part of government and for parliament trust has necessarily another meaning, and, while it may contain neutral elements such as politicians handling crises competently, partisan aspects are part of it. For instance, politicians are expected by their constituencies to do what they promised to do at elections. This will be valued by their constituencies but not by other voters. As a consequence it is only logical that trust in government and parliament cannot reach the level of that of the judiciary, as long as the judiciary is not politicized itself. This is borne out by the data. In all well-established democracies trust in the judiciary is higher than in parliament and government. Thus, while at first sight equality of trust in the three powers of the state would seem desirable from a perspective of balance, equality cannot be the goal. For the judiciary an aspiration level would be to score higher than the public administration. In the mean across countries this is realized.

## Alignment in Groups of Countries

Ordering the countries by means of one of the variables, trust in the (national) judiciary, three groups of countries are selected to examine the interaction of the perception variables at different levels of performance: the lowest 20%, the highest 20% and 20% symmetric around the median. Table 7.1 presents the results. In the lowest group trust in the judiciary is at the level of the other powers of the state, and is far below trust in public administration and regional and local public authorities. In the countries concerned trust in the judiciary at the EU-level is much higher than in the judiciary at the national level, but this does not seem to give the ECJ the trust that makes it politically risky for national government and parliament to ignore its judgments (see also Chapter 10.1007/978-3-030-63143-7_2). In these countries judges and lawyers are positive about judicial independence, but the general public is not. Neither are the parties. Citizens with a high education are even more negative about independence than citizens with a low education. This is remarkable. Citizens with a low education may be more susceptible to influence of the other state powers that in these countries show little respect for judicial independence. Not only respect by government and parliament is low, also in the court room respect by parties and lawyers is often not present.^1^ This group of countries provides a clear case of the judiciary being disconnected from society. The situation of these countries seems to be stable in a negative equilibrium, as there are no strong forces that pull towards judicial independence.

**Table 7.1:** Trust, perceived independence and respect for independence for three groups of countries

	Lowest trust	Middle trust	Highest trust
*Trust by general public (% that tends to trust) in:*			
National judiciary	26.6%	49.6%	80%
Average parliament and government	24.2%	33.7%	60.8%
Average public administration and regional/local authorities	37.9%	52.1%	71.0%
European Court of Justice	44.8%	46.8%	65.6%
*Perceived judicial independence (score 0–10) by:*			
Judges (about independence of all judges)	7.1	8.0	9.1
Lawyers	5.9	6.0	8.0
General public	3.9	5.5	7.3
General public high education	3.8	5.6	7.6
General public low education	4.3	5.2	6.4
Parties	3.6	5.2	6.8
*Respect for judicial independence perceived by judges (score 0–10) of:*			
Court users (average parties and lawyers)	6.5	7.4	8.6
Political system (average parliament, government and media)	5.2	5.8	7.5
Court management	7.7	8.2	8.9

*Notes*

Lowest 20%: BG, HR, LT, SK, SL

Middle 20%: BE, CZ, EL, HU, LT

Highest 20%: AT, DK, FI, NL, SW

At the other end of the spectrum, nearly all variables point to a stable equilibrium as well, but then at a high level of trust and independence. In terms of trust, the courts outperform not only the other state powers but also the less-political public sector institutions. In addition, the link with the EU is relatively strong. Even if a government would be tempted to ignore judgments of the ECJ, this would likely meet resistance from within society, and could be risky for the government. Perceptions of independence by the respective groups are all positive. However, the perceptions of independence by parties and citizens with a low education are not as positive as the other outcomes would lead to expect. As a result, also in these countries the difference between judges and the general public and, in particular, the part that has little education is large. Thus, even the best performing judiciaries are not aligned with society. While the judiciary plays its role in a capable manner, its high self-esteem may cause it to be complacent, and as a result not deeply interested in the court users or the developments society is undergoing. This complacency shows in the lack of court user surveys (only two of the five countries in this group conduct such surveys) and, for instance, slow adaptation to digital and on-line communication, as a result of which courts also in these countries were cornered by the COVID 19 pandemic (ELI 2020).

In the middle, there is a diverse range of countries that seem to be in a less stable situation. Trust in the judiciary is well above trust in the other two branches of government, but just falls short of the public administration, and it is in an absolute sense low. The link with the European level is weak, and does not differ much from the lowest group. Perceived independence by citizens with low education and parties is only marginally positive, and differs starkly from the views of the judges. Respect for independence by the political system is low. These countries are likely to be evolving, either positively or negatively. Hungary and, before the detrimental reforms, Poland are cases in point of negative evolution, while Lithuania shows a positive evolution.

**Fig. 7.1:**
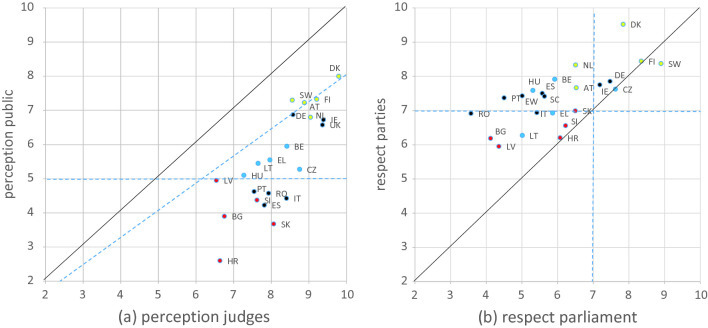
Alignment of judiciary and society, average scores per country **a** Perception of independence by judges and public. **b** Respect for independence by parliament and parties to lawsuits. Red dots: lowest 20% of trust in judiciary, blue: 20% around the median and yellow: highest 20% (*Note* See list of country abbreviations at the end of Chapter 10.1007/978-3-030-63143-7_3) (Color figure online)

Figure 7.1a and b depict the essential data for all countries individually. If a difference smaller than 20% in perceived independence by judges and public is required for a judiciary to be aligned with society (see the dashed line in Fig. 7.1a), preciously few judiciaries live up to that standard. A combination of positive perceptions by judges and negative perceptions by the public occurs frequently. This happens in 9 countries.^2^ In addition to the countries in the lowest 20% group, this occurs in Italy, Portugal, Spain and Romania. Figure 7.1b addresses respect for independence, and exhibits results for two actors, one from the category of court users (parties) and the other from the political system ( parliament). The scores reflect the percentages of judges that feel their independence is (not) respected. It is to some extent arbitrary at which score the independence of the judiciary is seen to be not respected anymore. In the figure demarcation lines are drawn at a score of 7, roughly corresponding with 70% of the judges feeling respected. Using this criterion, few countries are fully in the clear, in particular with regard to respect from parliament.

## Causes of Disconnect and Lack of Alignment

Can disconnect and lack of alignment be attributed to general causes? Two scenarios were suggested in Chapter 10.1007/978-3-030-63143-7_3. The alternative storylines were—briefly summarized—either the judiciary being overly confident or society overly critical. To review the evidence collected so far, in Chapter 10.1007/978-3-030-63143-7_3 the comparison of citizens and companies with and without experience with disputes at the courts was examined. For citizens the data show a lower appreciation of independence by citizens with than without experience. This does not bode well for the judiciary. However, it was also shown that views become more extreme, suggesting in particular that parties that lose their cases tend to have a (more) negative appreciation of independence. In combination with the general argument that going to court is not a positive experience as parties have to surrender control, it seems that the perceptions of the parties are overly negative. In Chapter 10.1007/978-3-030-63143-7_4 it was concluded that lay judges have similar perceptions about independence as professional judges. Albeit for a relatively small number of countries, the perceptions of lay judges give credence to the perceptions of judges.

From the data about the respect for independence it can be concluded that in the mean a large majority of judges feels their independence respected by the court users (see Fig. 7.1b for the parties to lawsuits). This as well as the respect for independence by court leaders is consistent with the positive perception of independence by judges. Judges experience less respect by the political system including the media. Thus, at system level there are more tensions than at the day-to-day work in the courts. The opinion of the general public is likely to be affected by media and politics. In particular if judges focus on their daily work as they are prone to do and which is where independence eventually materializes, they have the arguments on their side to be more positive about judicial independence than the general public. To conclude, judiciary and society are generally not aligned and in several countries there is an outright disconnect, but the judiciary is substantially more independent that the general public realizes. We conclude that both story lines are needed to explain the observed differences, but the relative contribution of both is difficult to assess.

## Consequences of Disconnect and Lack of Alignment

Disconnect and lack of alignment may reduce the willingness of parties to bring cases to court. One would expect that in these circumstances plaintiffs in disputes more often either give up their claims altogether or try to find alternatives that are more trustworthy. Of course, they would need to convince defendants to agree with such a course. This is often difficult to achieve. At the other end of the spectrum, it is likely that independent courts that are not swayed by specific interests of any kind, are more consistent in their interpretation of the law, and their judgments can be predicted accurately. Therefore, it would be relatively easy to convince defendants to negotiate out of court and to settle, and there would be no reason to go to court.

The most relevant type of case to examine here are civil cases, as these cases leave choice to the parties and have some homogeneity across jurisdictions. To give a first impression of the data, as gathered by CEPEJ, the countries in the 20% lowest group of trust have 2.9 civil litigious cases per 100 inhabitants, and the highest group 0.68 civil cases.^3^ It seems that in the countries that experience a disconnect people go to court abundantly, despite their misgivings about the independence of the judiciary. The same phenomenon has been observed for Russia (Hendley 2013). These figures do not prove, of course, that a disconnect leads to high case load, as many other variables play a role. Other factors such as disposition time and availability of alternatives, but also cultural factors like conflict handling behaviour need to be controlled for. For the present analysis it has to suffice that a disconnect does not dominate the actual use of the civil courts. The data of CEPEJ on administrative law show an inverted pattern with 0.22 cases per 100 inhabitants for the 20% lowest group versus 0.76 cases for the 20% highest bracket.^4^ This suggests that in these countries people avoid litigating against the state. However, the scope of administrative law differs among countries, and an in-depth analysis is needed before firm conclusions can be drawn. That there are issues in administrative law or more in general with cases against government, is shown in the latest judges survey of the ENCJ (2019). The survey includes the statement that judgments that went against the interests of the government were usually implemented/enforced in the country of the respondent. In the lowest bracket of trust 23% does not agree with the statement and 52% is not sure, while in the 20% highest bracket 12% does not agree and 31% is not sure.^5^ Thus, when one wins a case against the government, the chances that the government will honour the judgment are much lower in the former than in the latter countries. This reduces the incentive to go to court. This issue is broader than the lowest bracket, as in the middle group these percentages were 25% and 43%.

At the system level, lack of alignment and disconnect, in particular, weaken the position of the judiciary in the trias politica, and this may have diverse consequences ranging from insufficient allocation of resources, politicized appointments to governance bodies of the judiciary, taking tasks away from the judiciary and, as just noted, refusal by government to implement judgments. A disconnect may also make it easier for governments to succeed in policies towards illiberal democracy, including reforms of judiciaries to bring them under the control of government or parliament. After all, governments do not have to fear large popular support for the judiciary and its independence. In the multi-level governance system of the EU, resistance will come from the EU institutions and currently, in particular, from the ECJ that, as was shown, is trusted more than national judiciaries, but probably not enough to have decisive influence.

A disconnect but also a milder lack of alignment is likely to be self-perpetuating, as judiciaries do not see much need for connection with society, accountability, change and innovation, while society gets more frustrated by the lack thereof. To illustrate diverging sense of urgency, in the recent surveys among judges and lawyers it is found, for instance, that on the question whether judicial corruption is effectively addressed by the judicial authorities in the respondent’s country a large difference exists between what judges and what lawyers answer.^6^ For the 20% lowest bracket of trust, 12% of judges and 53% of lawyers give a negative answer and for the 20% highest bracket 2% of judges and 14% of lawyers. This does not show much recognition among judges of the urgency of change seen by lawyers, in particular, in the lowest 20% bracket.

## Judiciary in a Democracy

An important component of the lack of alignment and disconnect of the judiciary with society is the complicated relationship between the state powers. As discussed, many judges feel that their independence is not respected. The background of the tensions between the branches of government can be found in long term developments. The judiciary operates on the one hand at the micro level of the day to day adjudication of the many disputes involving citizens and/or businesses, and on the other hand at the macro level of fundamental decisions that affect government, its responsibilities and the boundaries it cannot transgress. Because of these fundamental decisions, the way the judiciary is perceived and treated by the other state powers cannot be separated from the changes democracies in Europe are going through in their relation with the judiciary. Depending on the trend one focusses on, there are several perspectives on the development of European democracies. From a *political* perspective, the worldwide trend of the gradually increasing influence of the courts on political decision-making, so-called ‘ judicialization’, is particularly relevant. This long-term trend is generally interpreted as an ongoing transfer of power from the organs of representative democracy to judiciaries (see e.g. the review by Hirschl 2009). The increasing reach of constitutional courts is an important component of this trend, but also the regular administrative and civil courts have a growing impact on government policies. One of the multiple causes is the increasing importance of fundamental rights and freedoms and law in general. Constitutional democracy replaces popular democracy (Mény and Surel 2002): political decisions are subject to judicial review and the leeway of politicians is reduced. The development of the European Union with its far reaching regulations and creation of independent European and national regulators, is a major factor. As national judges enforce European law and in that sense can not only rhetorically be called European judges,^7^ the national judiciaries are seen as reducing the degrees of freedom of national politics. While politicians have committed themselves earlier to EU rules and regulations, the judiciary holds them to that commitment. Judicialization is part of a broader trend of “the rise of the unelected” (Vibert 2007). The agencification that was briefly discussed in Chapter 10.1007/978-3-030-63143-7_2 is part of this trend (Verhoest 2017). The ubiquitous creation of independent national regulators goes well beyond granting autonomy to agencies that remain under the direction of ministers. Their formal safeguards of independence are similar to those of the judiciary (OECD 2016), and they derive much of their position and power from the judiciary (OECD 2017). Another example of declining influence of national politicians is the position of the national Central Banks that in as far as community tasks (such as monetary policy) are concerned, are independent. Their independence has been arranged in a detailed manner, and forbids, for instance, the giving of instructions to the governors of the Bank. Recourse to a court is often possible if a Bank feels its independence is breached. The president of a national Central Bank can, for instance, appeal against his/ her dismissal to the ECJ, while other members of decisions making bodies can address a national court (ECB 2018).

These developments are experienced by politicians as a threat to representative democracy. In particular, populist politicians who oppose Europeanization regularly express fear of rule by judges (using terms such as government of judges, juristocracy, courtocracy, dikastocracy and kritocracy), but discomfort about loss of influence to the judiciary is broader among politicians than only the populist right and left. This judicialization happens at a time when representative democracy is under internal pressures, for instance due to the changing nature of political parties and their leadership (Papadopoulos 2013). In the context of this book, it should be noted that judicialization presupposes that the judiciary is ‘reasonably independent’ (Hirschl 2009, p 130). This brings us to the second perspective.

In a *rule of law* perspective, one can see the judiciary and in particular its independence under threat from politicians who have in many ways a strong grip on the judiciary and who do not want to relinquish that grip. Where this grip was relinquished in the past, politicians often yearn to get it back. If one examines the Justice Scoreboard of the EC and the indicators of independence and accountability of the ECNJ, the impression one gets of the judiciary is a conglomerate of judges that is in many respects dependent on the other state powers, even in some countries for the day to day management of the courts (EC 2020; ENCJ 2020; van Dijk and Vos 2018). Even in countries in which the judiciary is an autonomous organization, it is often heavily dependent on government and parliament for budget and for appointments to leadership positions, for instance, at Councils for the Judiciary (e.g. Torres Pérez 2019 on Spain). Pressure on the judiciary has also a public communication component: in the media the critique of lack of democratic legitimacy is often levelled against the judiciary when judgments are not welcome. In Central and Eastern European countries at the accession to the Union the independence of the judiciary was often well arranged, and this shows in the indicators of the ENCJ, mentioned above. These judiciaries have stronger formal safeguards than most countries in Western Europe that traditionally operate on the basis of mutual trust and respect. In several of the countries of Central and Eastern Europe politicians try to recapture the grip that they relinquished (Coman 2014; Kovács and Scheppele 2018; Sterk and van Dijk 2019). Their authoritarian leaders call the resulting form of government illiberal democracy. Especially in these countries, judiciaries feel threatened. In other countries there is often a feeling within the judiciary of being kept short by the other state powers, also literally in terms of caseloads and budgets. The judiciaries of Europe brace themselves against an often combined power block of government and parliament to maintain their independence.

In a *access to justice* perspective, the focus is on the day-to-day plight of ordinary people and businesses seeking justice. In all countries a substantial part of their legal needs are not met (for a brief summary of the extensive literature see OECD/WJP). If disputes have been resolved by settlement or adjudication, many believe this resolution was not achieved in a fair manner. In addition, in many judiciaries (larger) court cases take a long time, are costly and are administratively complicated, partly due to lagging ICT-systems. In this perspective the judiciary is an important part of conflict resolution chains which it ideally guides by a clear and uniform application of the law. As a result people would not have to go to court in most cases. Procedural justice plays, next to access to justice, a central role in this approach: not only in cases of citizens against government but in all party configurations. Procedural justice requires judges in each case to be independent and to convey this to the parties and, if the case is of sufficient interest, to the media and society in general.

Obviously, the first two perspectives collide, leading at best to misunderstandings between politicians who only see an empowered judiciary that they do not consider in need of strengthening further, and a judiciary that sees its—often already limited— independence under threat by reform proposals and by mundane issues like insufficient budgets. At its worst this leads to power struggles that spill over to the EU level. Government and parliament on the one side and the judiciary on the other side feel threatened by each other. The relatively low scores on respect for judicial independence by the political system, as perceived by the judiciary, testify to this from the perspective of the judiciary. In this context, a beauty contest about who is trusted the most, is not helpful and, as was discussed in Chapter 10.1007/978-3-030-63143-7_6, beside the point. One may wonder what is the way out of this situation.

In this Chapter the conclusion was reached that judiciaries are characterized by a lack of alignment or even a disconnect with society. This shows in particular in very diverse views of judges and citizens about judicial independence, and in case of a disconnect by positive perceptions of independence by judges and negative perceptions by citizens, as well as low respect of independence by the court users and the political system. Lack of alignment and disconnect are to some extent a reflection of the above collision. Both are also connected to lack of knowledge in society about the judiciary which was shown by the much more positive perceptions of independence by knowledgeable lay judges than by ordinary (even highly educated) citizens. Judiciaries perform better than the public is aware of. Still, these reasons cannot fill the whole gap in perceptions between judges and the rest of society. Within the judiciary, the reason must be sought in a generally weak orientation on the court users. While many courts perform well in this respect by sticking to their core values, feedback mechanisms are hardly anywhere established. In Chapter 10.1007/978-3-030-63143-7_3 the weak court user orientation that speaks from the scarcity of court user surveys was discussed. Guaranteeing that people get real access to justice, irrespective of their background and that of the other parties in judicial proceedings, is not at the forefront in most courts. This affects the appreciation of the public for the courts and can easily lead to a negative perception of independence and low trust. In addition, it weakens the position of the judiciary in the debate with politicians, as judges cannot count on much support from the population.

Therefore, the third perspective of access to justice is important for society and judiciary. This perspective as such is likely to be less controversial than the other perspectives, despite its reliance on the rule of law in every-day cases. It requires constructive co-operation within conflict resolution chains in order to empower people to resolve disputes themselves on the basis of adequate information or assistance, and, secondary, to unburden the courts of cases that are trivial from a legal point of view. To let such chains work smoothly, courts have to be consistent and fast. And they must be truly independent, and make sure that the parties and everybody else involved are fully aware of that. In this way, the judiciary will better connect with society, and it will, indirectly, promote its independence. Building broad public support by promoting and guaranteeing access to justice, including fair trial, is not only essential to maintain the authority of the judge in the court room, but also to maintain the position of the judiciary in the political arena.

However, the positive perception of their independence by most judges that emerged from the surveys, leads inadvertently to a low urgency to improve performance and image. This rather myopic attitude in a further broad minded group of professionals is one of the striking characteristics of the judiciary (see for the US Rottman and Tomkins 1999). As mentioned already, it would be helpful if judges became more interested in the experiences of the court users, in particular with regard to fundamental issues as independence and impartiality. A far sighted approach would to be to try and bring the perceptions of the court users and citizens in general together with those of the judges. Exactly in view of the importance of avoiding the appearance of dependence and partiality, the reduction of differences in perceptions is advisable, even if this means adjusting the perceptions of the judges downward.

Returning to the interaction of the powers of the state, it was suggested earlier in this Chapter, based on the data presented, that the outcome may or may not be that the judiciary of a country is in a stable equilibrium. A stable equilibrium seems to occur at a high or at a low level of—in combination—perceived independence, respect for independence and trust in the judiciary. If a stable system is out of equilibrium for instance due to temporary negative events such as a deep economic crisis or positive events such as large external support, it will return after some time to its former equilibrium. The judiciaries that were classified as stable are not easy to get permanently out of balance. When in a positive balance, this is a desirable characteristic, but in a negative equilibrium, this is not. A disconnect between judiciary and society, that characterizes a negative equilibrium, is hard to overcome. In-between there are countries of which the powers of the state do not seem to be in equilibrium. Large improvements and large deteriorations in the rule of law occur and may even alternate. Poland and Hungary are examples of countries in which political leaders were able to change the judicial institutions radically, despite elaborate legal safeguards and despite being embedded in the European Union. These upheavals follow from the clash of perspectives that was discussed above. A long term defence against such upheavals is a sharp focus of the judiciary on delivering justice for the population, and to build popular support.

## Footnotes

1 The score in Table 7.1 for respect by court users in the lowest trust group corresponds to 57% of the responding judges (strongly) agreeing that their independence is respected, 16% disagreeing and 27% is uncertain.

2 Positive: the score is larger than 5, Negative: the score is smaller than 5. The smallest difference between a positive score by judges and a negative score by citizens is 23% (1.5 point).

3
CEPEJ (2018, Fig. 5.5) (no data on Bulgaria).

4
CEPEJ (2018, Fig. 5.23).

5
ENCJ (2019, Fig. 10).

6
ENCJ (2019, Fig. 34) and ENCJ/ CCBE (2019, Fig. 20).

7 E.g., “National judges are European judges and they oversee the application of EU law”. Vice-president of the EC, Timmermans, on Twitter 21 February 2019.
